# Metformin and Dichloroacetate Suppress Proliferation of Liver Cancer Cells by Inhibiting mTOR Complex 1

**DOI:** 10.3390/ijms221810027

**Published:** 2021-09-17

**Authors:** Tae Suk Kim, Minjong Lee, Minji Park, Sae Yun Kim, Min Suk Shim, Chea Yeon Lee, Dae Hee Choi, Yuri Cho

**Affiliations:** 1 Department of Internal Medicine, Kangwon National University Hospital, Kangwon National University School of Medicine, Chuncheon 24341, Korea; greatstone@kangwon.ac.kr (T.S.K.); qnwia@naver.com (C.Y.L.); dhchoi-md@kangwon.ac.kr (D.H.C.); 2 Department of Internal Medicine, Ewha Womans University College of Medicine, Seoul 07804, Korea; jewelbox6@hanmail.net; 3 Department of Internal Medicine, Ewha Womans University Medical Center, Seoul 07804, Korea; 4 Department of Internal Medicine, CHA Gangnam Medical Center, CHA University School of Medicine, Seoul 06135, Korea; 5 Department of Pediatrics, College of Medicine, The Catholic University of Korea, Seoul 06591, Korea; sysmile@gmail.com; 6 Division of Bioengineering, Incheon National University, Incheon 22012, Korea; msshim@inu.ac.kr; 7 Center for Liver and Pancreatobiliary Cancer, Research Institute and Hospital, National Cancer Center, Goyang 10408, Korea

**Keywords:** liver cancer, Warburg effect, mTOR complex I, REDD1

## Abstract

The Warburg effect is important for cancer cell proliferation. This phenomenon can be flexible by interaction between glycolysis and mitochondrial oxidation for energy production. We aimed to investigate the anticancer effects of the pyruvate dehydrogenase kinase inhibitor, dichloroacetate (DCA) and the mitochondrial respiratory complex I inhibitor metformin in liver cancer cells. The anticancer effect of DCA and/or metformin on HepG2, PLC/PRF5 human liver cancer cell lines, MH-134 murine hepatoma cell lines, and primary normal hepatocytes using MTT assay. Inhibition of lactate/ATP production and intracellular reactive oxygen species generation by DCA and metformin was investigated. Inhibition of PI3K/Akt/mTOR complex I was evaluated to see whether it occurred through AMPK signaling. Anticancer effects of a combination treatment of DCA and metformin were evaluated in HCC murine model. The results showed that metformin and DCA effectively induced apoptosis in liver cancer cells. A combination treatment of metformin and DCA did not affect viability of primary normal hepatocytes. Metformin upregulated glycolysis in liver cancer cells, thereby increasing sensitivity to the DCA treatment. Metformin and DCA inhibited mTOR complex I signaling through upregulated AMPK-independent REDD1. In addition, metformin and DCA increased reactive oxygen species levels in liver cancer cells, which induced apoptosis. A combination treatment of metformin and DCA significantly suppressed the tumor growth of liver cancer cells using in vivo xenograft model. Taken together, the combined treatment of metformin and DCA suppressed the growth of liver cancer cells. This strategy may be effective for patients with advanced liver cancer.

## 1. Introduction

In early stage of hepatocellular carcinoma (HCC), various treatment options can be considered such as radiofrequency ablation, resection, radiotherapy, and transplantation [1,2]. However, in advanced stage of HCC, target therapy is only recommended: current anti-angiogenic drugs, such as sorafenib, regorafenib, lenvatinib, and cabozantinib, significantly prolong survival of patients with advanced HCC [3]. 

Recently, it was emphasized that magnetic resonance imaging (MRI) can be helpful to detect HCC within early stages [4,5,6,7,8,9]. Particularly, in obese or cirrhotic patients, sensitivity of ultrasound (US) for detection of HCC within early stages can be problematic due to poor sonic window [6,10,11,12,13,14,15]. However, in real field, US exams are the main surveillance tool because of unsolved medical insurance problems for high cost MRI. Unfortunately, 30–35% of HCC is detected at an advanced stage [6,7]. In HCC patients with advanced stage, the long-term survival benefit from currently used anticancer treatments is a modest improvement of three months, which is far from satisfactory [6,7,16,17]. Clinically, there are critical limitations to treat HCC patients: (1) a lack of available drugs after failure of tyrosine kinase inhibitors [18,19]; (2) increasing need of target therapy agents in patients with intermediate stage who showed refractoriness for transarterial chemoembolization or inadequate safety margin of embolized area after TACE [12,17,20,21,22,23,24]; (3) substantial risk of HCC recurrence even after five years in patients who underwent curative resection [25,26,27]; (4) no available drugs for target therapy in patients with decompensated cirrhosis [28]. Therefore, there is a need to explore novel strategies as alternatives to the currently used drugs in patients with advanced HCC.

Recently, it was reported that inhibition of energy production signaling such as PI3K/Akt suppressed epithelial-to-mesenchymal transition in hepatocellular carcinoma through the Snail/GSK-3/beta-catenin pathway, which describes a therapeutic approach to suppress the development of HCC employing energy blockers [29,30,31]. Among various energy blockers, DCA, an inhibitor of pyruvate dehydrogenase kinase (PDK), promotes oxidative metabolism by activating the pyruvate dehydrogenase complex (PDH) and subsequent flux of glucose carbon through the tricarboxylic acid cycle [32]. PDK is upregulated in a number of cancers and DCA reverses the glycolytic phenotype resulting from its enhanced activity [32]. A consequence of DCA-induced oxidative metabolism is the production of reactive oxygen species (ROS), and this enhanced oxidative stress promotes death of cancer cells [33,34]. Recent evidence suggests that the efficacy of DCA is enhanced by the anti-diabetic drug metformin [35,36]. Metformin, a cationic biguanide, readily accumulates in the mitochondria, where it inhibits complex I of the electron transport chain (ETC) [37]. Inhibiting the ETC induces energetic stress that promotes activation of adenosine monophosphate kinase (AMPK), subsequently leading to catabolism that restores energetic homeostasis in cancer cells [38]. Metformin also suppresses tumor growth by inducing cycle arrest, promoting apoptosis, and suppressing autophagy [39]. Given their potential mutual compensatory effects in terms of energy production, we aimed to uncover whether metformin and DCA can effectively kill liver cancer cells to enhance each cytotoxicity. We also tried to characterize the mechanism of anticancer effects of metformin and DCA in liver cancer cells.

## 2. Results

### 2.1. Metformin and DCA Effectively Induced Apoptosis in Liver Cancer Cells

**Figure 1:**
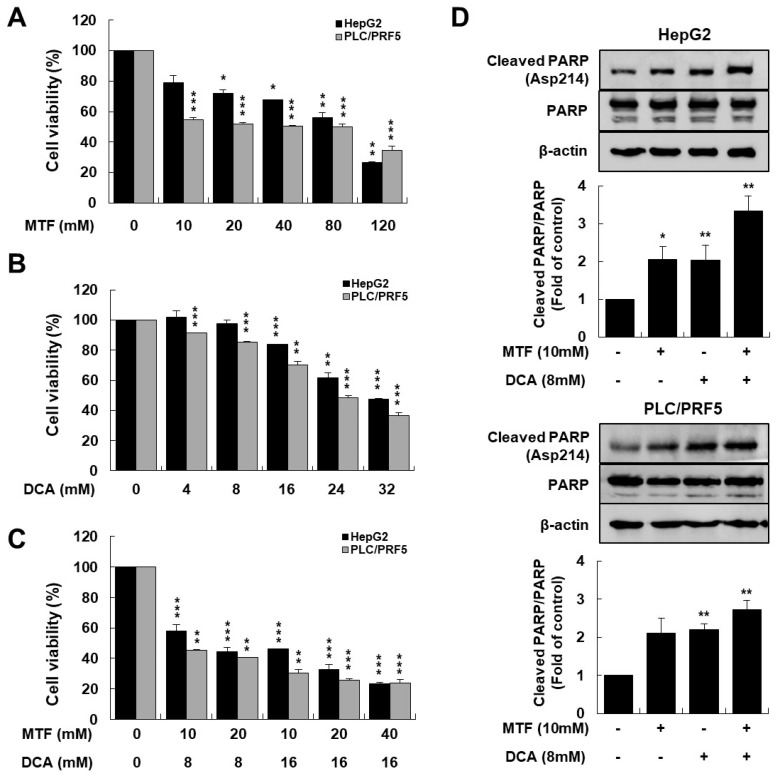
Metformin and DCA effectively induced apoptosis in liver cancer cells. HepG2 and PLC/PRF5 cells were treated with the indicated concentrations of metformin and DCA for 24 h. (**A**) Cytotoxicity of metformin on liver cancer cells significantly increased at a higher concentration compared to the control. (**B**) Cytotoxicity of DCA on liver cancer cells significantly increased at a higher concentration compared to the control. (**C**) Cytotoxicity of metformin and DCA on liver cancer cells significantly increased at higher concentrations compared to the control: the combination index values for the combination of metformin and DCA were 0.63 and 0.58 for HepG2 and for PLC/PRF5 cells, respectively. (**D**) Expression of cleaved PARP significantly increased when liver cancer cells were treated with metformin and DCA compared to each treatment alone. The bottom panel shows relative band density. * *p* < 0.05; ** *p* < 0.01; and *** *p* < 0.001.

To investigate whether there is the anticancer effect between metformin and DCA in suppressing the growth of liver cancer cells, HepG2 and PLC/PRF5 cells were treated with metformin, DCA, or both (Figure 1A,B). Co-treatment with metformin and DCA significantly suppressed growth of the liver cancer cells compared to metformin or DCA alone, respectively (*p* < 0.001 in HepG2 and *p* < 0.01 in PLC/PRF5, respectively; Figure 1C), and the combination of 10 mM metformin and 8 mM DCA inhibited cell viability of 50% (IC_50_ values) compared to the control. 

The cytotoxic effect of the combined metformin and DCA treatment on HepG2 and PLC/PRF5 cells was evaluated using combination index (CI) using the Chou and Talalay: CI = (dA/DA) + (dB/DB), where dA and dB are the IC_50_ value of compound A and B in combination, and DA and DB are the IC_50_ values of a single compound A or B, respectively. CI values of <1, 1, and >1 indicate synergism, additivity, and antagonism in combined agent action, respectively [40]. There was a potent anticancer effect of metformin and DCA on inhibiting liver cancer cell growth because the CI values for the combined metformin and DCA treatment were 0.63 and 0.58 for HepG2 and PLC/PRF5 cells, respectively. Metformin and DCA effectively induced apoptosis in liver cancer cells. Cleaved PARP (a marker of apoptosis) expression was potentiated in liver cancer cells co-treated with metformin and DCA compared to those treated with metformin or DCA alone (Figure 1D). When HepG2 and PLC/PRF5 cells were treated with the combination of 10 mM metformin and 8 mM DCA for 24 h in annexin V-FITC staining, the proportion of apoptotic cells was significantly higher as compared to the control (all *p* < 0.001; Supplementary Figure S1).

**Figure 2:**
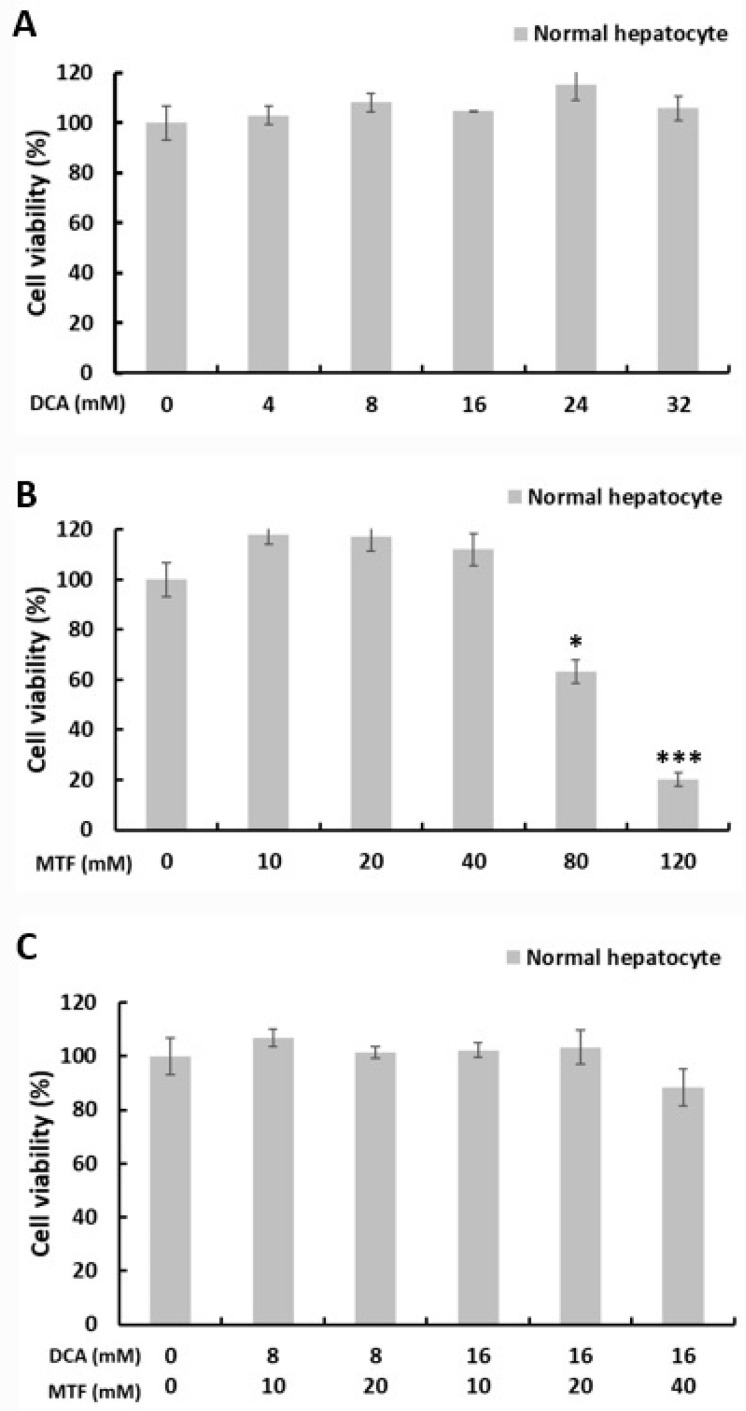
Co-treatment with metformin and DCA did not show cytotoxicity in primary normal hepatocytes. Primary normal hepatocytes were treated with the indicated concentrations of metformin and DCA for 24 h. (**A**) There was no cytotoxicity of DCA on primary normal hepatocytes even at high concentrations compared to the control. (**B**) At low concentrations of metformin (≤40 mM), there was no significant cytotoxicity of metformin on primary normal hepatocytes; at high concentrations of metformin (≥80 mM), cytotoxicity of metformin on primary normal hepatocytes significantly increased compared to the control. (**C**) Co-treatment with metformin and DCA did not show cytotoxicity in primary normal hepatocytes compared to the control. * *p* < 0.05 and *** *p* < 0.001.

In contrast, at each IC_50_ value of 8 mM DCA (Figure 2A) or 10 mM metformin (Figure 2B) for liver cancer cells, there was no significant cytotoxicity to primary normal hepatocytes. Co-treatment with metformin (10 mM) and DCA (8 mM) did not show cytotoxicity to primary normal hepatocytes (Figure 2C). However, at high concentrations of 80 and 120 mM metformin, we observed cytotoxic effects of metformin on primary normal hepatocytes.

### 2.2. Metformin Sensitized Liver Cancer Cells to DCA through Increasing Glycolysis

**Figure 3:**
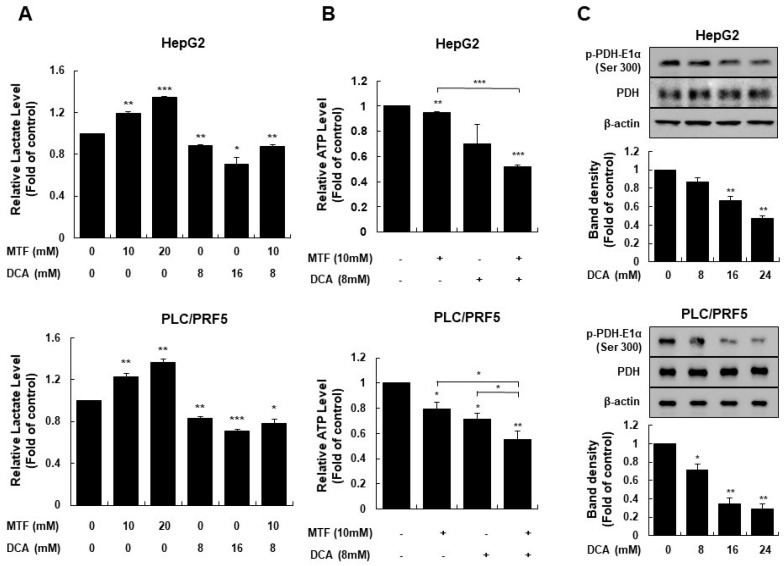
Metformin sensitized HepG2 and PLC/PRF5 cells to DCA by increasing glycolysis. Intracellular ATP production in liver cancer cells was suppressed by the metformin and DCA co-treatment. (**A**) Extracellular lactate levels increased significantly at higher metformin concentrations and decreased at higher DCA concentrations in liver cancer cells. The metformin and DCA co-treatment significantly suppressed lactate production compared to the control in liver cancer cells. (**B**) Intracellular ATP levels significantly decreased when liver cancer cells were treated with metformin or DCA, and co-treated with metformin and DCA, respectively. The metformin and DCA co-treatment significantly suppressed lactate production compared to the single treatment of metformin or DCA alone, and the control. (**C**) DCA significantly suppressed phosphorylation of the pyruvate dehydrogenase complex compared to the control in a dose-dependent manner. * *p* < 0.05; ** *p* < 0.01 and *** *p* < 0.001.

Metformin has been well-known to inhibit respiratory complex I of the ETC in the mitochondria [37]. Inhibiting mitochondrial respiratory complex I induces energetic stress that promotes activation of AMPK, subsequently leading to catabolic metabolism that restores energetic homeostasis [38]. To elucidate whether inhibiting complex I with metformin induces glycolysis and depletes intracellular ATP in liver cancer cells, extracellular lactate levels, and intracellular ATP levels were measured. Metformin increased lactic acid production in a dose-dependent manner in liver cancer cells (*p* = 0.007 in HepG2, *p* = 0.01 in PLC/PRF5 for 10 mM metformin and *p* < 0.001 in HepG2, *p* = 0.002 in PLC/PRF5 for 20 mM metformin; Figure 3A), but decreased intracellular ATP levels in liver cancer cells (*p* = 0.02 in HepG2, *p* = 0.03 in PLC/PRF5; Figure 3B). 

As phosphorylation of the PDH complex is associated with the Warburg effect, we sought to determine if the DCA treatment alters lactate production in liver cancer cells [41]. A 24-h incubation with 8 mM DCA resulted in a significant reduction of lactate compared to the control, suggesting a shift towards glucose oxidation (*p* = 0.07 in HepG2, *p* = 0.02 in PLC/PRF5 for 8 mM DCA and *p* = 0.008 in HepG2, in *p* = 0.005 in PLC/PRF5 for 16 mM DCA; Figure 3A). The DCA treatment reduced phosphorylation of the E1α subunit of the PDH complex in a dose-dependent manner following a 4 h incubation (Figure 3C), thereby DCA inhibiting lactate production in liver cancer cells. DCA also decreased intracellular ATP levels in liver cancer cells (*p* = 0.11 in HepG2 and *p* = 0.02 in PLC/PRF5 for 8 mM DCA; Figure 3B).

Co-treatment with DCA and metformin significantly suppressed lactic acid production and intracellular ATP production in liver cancer cells compared to the control (*p* = 0.005 in HepG2, *p* = 0.02 in PLC/PRF5 for suppression of lactate production, and *p* < 0.001 in HepG2, *p* = 0.01 in PLC/PRF5 for suppression of ATP production; Figure 3A,B). 

### 2.3. Metformin and DCA Effectively Suppressed mTORC1 Expression by Inhibiting PI3K/Akt/mTORC1 Signaling and Upregulating REDD1

**Figure 4:**
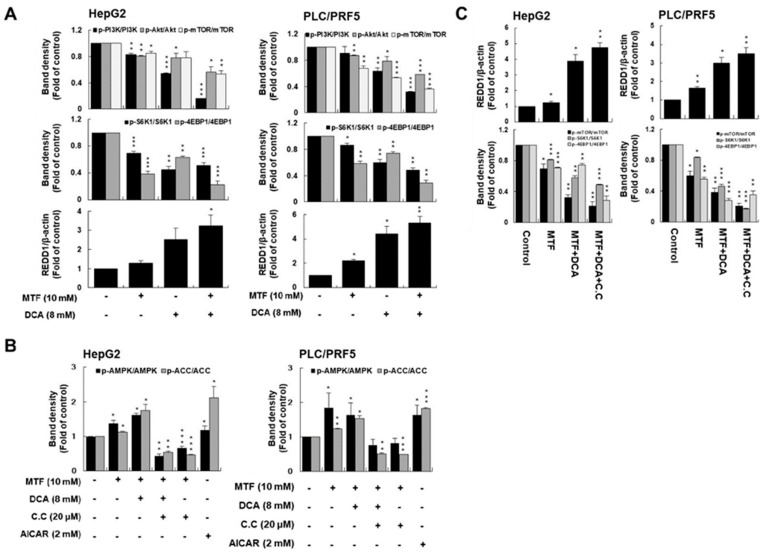
Cells were exposed to 10 mM metformin, 8 mM DCA, or both for 24 h. (**A**) Co-treatment with metformin and DCA significantly suppressed PI3K/Akt/mTORC1 signaling in liver cancer cells compared to the control. Co-treatment with metformin and DCA also significantly suppressed expression of the mTORC1 effectors S6K1 and 4EBP in liver cancer cells compared to the control. (**B**) Co-treatment with metformin and DCA stimulated p-ACC expression as an AMPK effector in liver cancer cells compared to the control. Compound C as an AMPK inhibitor suppressed p-ACC expression when treated with metformin and DCA. AICAR, an AMPK activator, potentiated p-ACC expression. (**C**) Metformin and DCA effectively induced REDD1 expression. Upregulation of REDD1 expression by metformin and DCA was not affected by compound C (the AMPK inhibitor). Regardless of inhibition of AMPK expression, co-treatment with metformin and DCA downregulated the mTORC1 effectors S6K1 and 4EBP in liver cancer cells through upregulation of REDD1 expression. * *p* < 0.05; ** *p* < 0.01; and *** *p* < 0.001.

It has been well-known that mTORC1 is one of the key molecules in liver cancer carcinogenesis [42]. We assessed whether co-treatment with DCA and metformin effectively inhibited PI3K/Akt/mTORC1 signaling. Treatment with metformin or DCA suppressed the expression of PI3K/Akt/mTORC1 signaling in liver cancer cells. Co-treatment of metformin and DCA significantly suppressed PI3K/Akt/mTORC1 signaling compared to the control (*p* < 0.001 for p-PI3K/PI3K, *p* = 0.02 for p-Akt/Akt, *p* = 0.005 for p-mTOR/mTOR in HepG2 cells and *p* < 0.001 for p-PI3K/PI3K, *p* < 0.001 for p-Akt/Akt, *p* < 0.001 for p-mTOR/mTOR in PLC/PRF5 cells, respectively; Figure 4A). Regarding effectors of mTORC1 such as S6K1 and 4EBP1, co-treatment of metformin and DCA significantly suppressed S6K1 and 4EBP1 signaling compared to the control (*p* < 0.001 in HepG2 cells and PLC/PRF5 cells; Figure 4A and Supplementary Figure S2).

Given that metformin inhibited mTORC1 activity in cancer cells through energy stress to activate AMPK [43], we also assessed whether co-treatment with metformin and DCA affected mTORC1 expression by activating AMPK expression. 

Co-treatment of metformin and DCA significantly potentiated AMPK expression as compared to the control: co-treatment of metformin and DCA increased expression of p-ACC as an effector of AMPK signaling as compared to the control (*p* = 0.03 in HepG2, *p* = 0.01 in PLC/PRF5; Figure 4B). However, compound C known as an AMPK inhibitor (20 μM) inhibited expression of p-ACC when treated with metformin and DCA in both cell lines; AICAR as an activator of AMPK (2 mM) potentiated expression of p-ACC as compared to the control (*p* = 0.006 in HepG2, *p =* 0.004 in PLC/PRF5 for compound C and *p* = 0.04 in HepG2, *p* < 0.0001 in PLC/PRF5 for AICAR; Figure 4B and Supplementary Figure S2).

Although co-treatment of metformin and DCA activated AMPK signaling, inhibition of mTORC1 expression was not mediated by AMPK expression, but by REDD1 expression. Considering that energetic stress induces REDD1 expression in cancer [44], we assessed whether energetic stress induced by co-treatment with metformin and DCA affected REDD1 expression. REDD1 is known to interact with TSC2 and to increase the inhibitory effect of the TSC1/TSC2 heterodimer on mTORC1 activity [45]. We found potent anticancer effects of metformin and DCA on the induction of REDD1 expression (Figure 4C and Supplementary Figure S2). However, induction of REDD1 expression by metformin and DCA was not affected by compound C. We concluded that the induction of REDD1 by metformin and DCA contributes to the inhibition of mTORC1 activity in an AMPK-independent manner.

### 2.4. Modulating ROS Is Important for Anticancer Effects of the Metformin and DCA Treatment

**Figure 5:**
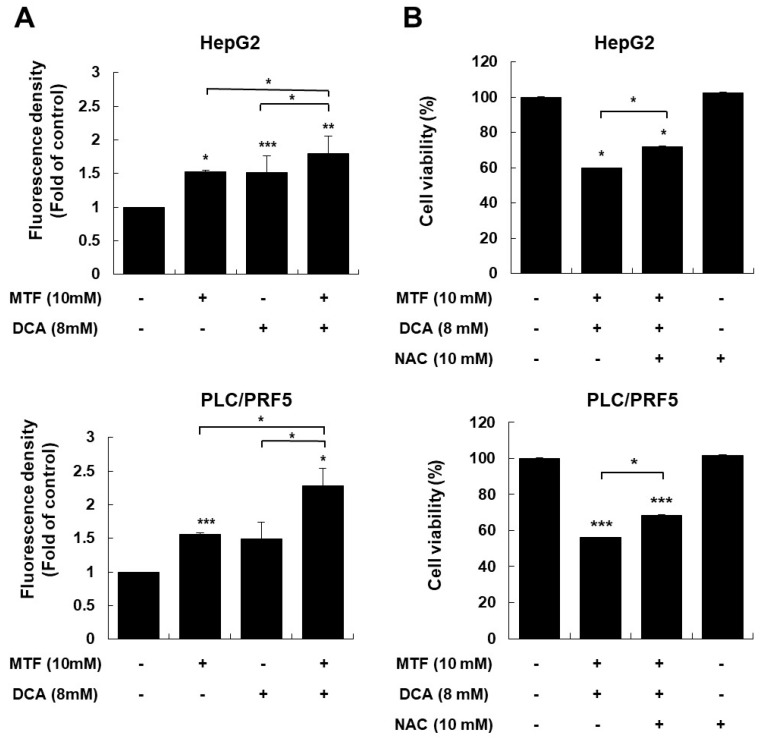
Co-treatment with metformin and DCA increased oxidative stress in HepG2 and PLC/PRF5 cells. Liver cancer cells were treated with metformin, DCA, or both for 1 h. (**A**) Co-treatment with metformin and DCA significantly increased reactive oxygen species (ROS) production in liver cancer cells compared to the control. (**B**) Treatment with the antioxidant N-acetylcysteine (NAC) (10 mM) significantly decreased the cytotoxic effects of the metformin and DCA co-treatment in liver cancer cells. * *p* < 0.05; ** *p* < 0.01; and *** *p* < 0.001.

Because glycolysis and oxidative metabolism are intrinsically linked to the generation of ROS [46], we evaluated whether activation of pyruvate dehydrogenase by DCA altered ROS production in liver cancer cells. Intracellular ROS levels in liver cancer cells in response to the DCA treatment significantly increased following a 1-h DCA treatment compared to the control (*p <* 0.001 for HepG2 and *p* = 0.07 for PLC/PRF5,Figure 5A), and by metformin compared to the control (*p* = 0.02 for HepG2 and *p <* 0.001 for PLC/PRF5, Figure 5A). Adding metformin further enhanced ROS production in the presence of DCA compared to metformin or DCA alone (*p* = 0.03 metformin + DCA vs. DCA, *p* = 0.03 metformin + DCA vs. metformin in HepG2 cells and *p* = 0.02 metformin + DCA vs. DCA, *p* = 0.04 metformin + DCA vs. metformin in PLC/PRF5; Figure 5A).

To clarify the association between increased ROS and cell death with DCA and/or the metformin treatment, we evaluated the anticancer effect of DCA and/or metformin when ROS levels in liver cancer cells were modulated. The antioxidant N-acetylcysteine significantly attenuated cytotoxicity of co-treatment with metformin and DCA (*p* = 0.03 in HepG2, *p* = 0.02 in PLC/PRF5; Figure 5B). This finding suggested that increased oxidative stress by co-treatment with metformin and DCA played an important role in the potent anticancer effect in liver cancer cells.

Mitochondrial ROS levels in HepG2 cells in response to metformin and DCA treatment increased significantly following a 1-h DCA treatment compared to the control (*p* < 0.001, Supplementary Figure S3). The measurement of reactive oxygen species (ROS) in tumor tissues was also performed based on the previous literature [47]. We used serum 4-hydroxynonenal (HNE) antibody (LSBio, Seattle, WA, USA) directed against antigens that can serve for a sensitive marker of increased oxidative stress. In tumor tissue, 4-HNE in the group treated with metformin and DCA was highly expressed (Supplementary Figure S3).

### 2.5. Anticancer Effect of a Combination Treatment of Metformin and DCA in Murine Models

**Figure 6:**
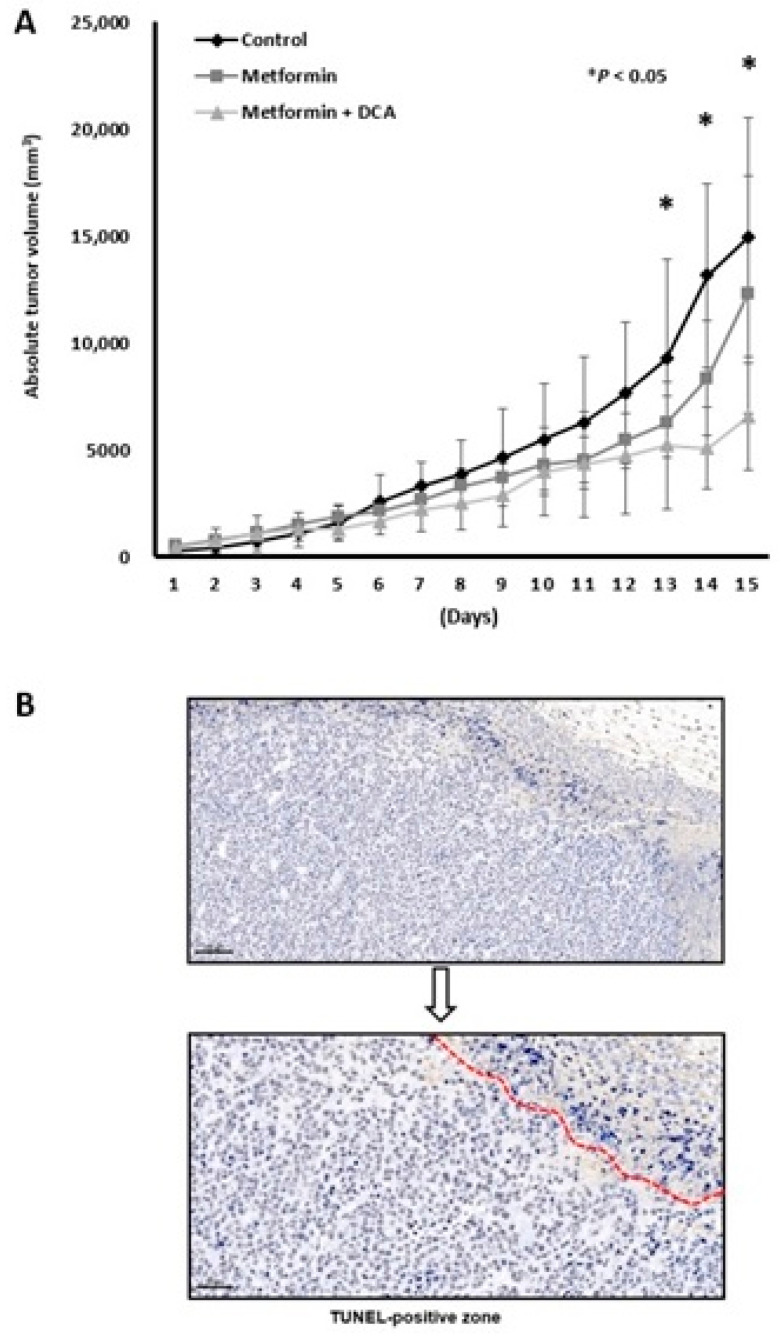
Anticancer effects of a combination treatment with metformin and DCA in a mouse model bearing MH-134 cells. (**A**) Tumor volume in the combination treatment group at 13–15 days was significantly smaller than that in the control or metformin treatment groups (all, *p* < 0.05). (**B**) In the combination treatment group, tumor tissues showed TUNEL-positive staining (400× magnification). * *p* < 0.05.

The anticancer effects of co-treatment with metformin and DCA were examined using an in vivo xenograft model. We started to treat mouse after tumor budding, whose size was over 200 mm^3^. It took six to eight days after transplantation of 5 × 10^7^ MH134 cells to mouse. There was no difference of periods from transplantation to tumor budding among three groups. Co-treatment with metformin and DCA significantly suppressed tumor growth compared to the control group at days 13, 14, and 15 after MH-134 tumor budding (Figure 6A; all, *p* < 0.05). Although the metformin treatment alone group tended to have lower tumor growth rates than the control group, there were no significant differences in tumor growth rates between the metformin treatment alone group and the control group. In the combination treatment group, dead cells were TUNEL-positive (Figure 6B). As compared to the control group, expression of cyclin D1, Ki-67 protein, and proliferating cell nuclear antigen was decreased in tumor tissues in the group treated with metformin and DCA was decreased (Supplementary Figure S4). The individual changes of tumor volume in the combination treatment group of metformin + DCA over time are suggested in Supplementary Figure S5.

## 3. Discussion

It is well-known that most tumors are characterized by the “Warburg effect”, whereby glycolysis is used for energy production even though oxygen levels are sufficient. Although energy metabolism of cancer cells is mainly dependent on the Warburg effect, some conditions can modulate the Warburg effect, such as inhibited mitochondrial oxidation or ROS status in cancer cells. We hypothesized that dual energy blockade of the Warburg effect and mitochondrial oxidation would effectively suppress growth of liver cancer cells. Dual energy blockade of glycolysis and mitochondrial oxidation might be important for death of cancer cells because cancer cells can use the energy factory of glycolysis or mitochondrial oxidation when one of the two is disadvantageous for survival. 

In this study, metformin accelerated lactate accumulation, which facilitated abnormal cancer-addicted aerobic glycolysis. DCA strongly inhibits the activity of PDKs and their downstream p-PDHE1α, leading to metabolic remodeling, reuse of oxidative phosphorylation, and lower lactate accumulation. However, reusing oxidative phosphorylation induced by DCA was also blocked by metformin treatment in liver cancer cells. Our results indicate that DCA sensitizes the anticancer effect of metformin by inhibiting the activity of PDKs, and metformin potentiated glycolysis in liver cancer cells. There was potent anticancer effect by co-treatment with metformin and DCA compared to that of each treatment alone. 

Anticancer effect of co-treatment with metformin and DCA can be explained by three mechanisms in this study: (1) potent depletion of the energy source, such as intracellular ATP, (2) inhibited mTORC1 singling, and (3) ROS-mediated cytotoxicity. The metformin and DCA co-treatment effectively suppressed ATP production by inhibiting glycolysis and mitochondrial oxidation in liver cancer cells, and thereby, it stimulated AMPK expression. The metformin and DCA co-treatment suppressed expression of mTORC1 via the PI3k/Akt/mTORC1 and REDD1 signaling pathways. Through activation of REDD1 expression, metformin and DCA had anticancer effects on mTORC1 activity as measured by S6K1 and 4EBP1 phosphorylation. Given that inhibition of AMPK expression by compound C did not affect inhibition of mTORC1 by metformin and DCA, the results suggested that the anticancer effect of metformin and metformin is AMPK-independent. 

We evaluated the anticancer effect of metformin and DCA on the induction of REDD1 expression to explain AMPK-independent inhibition of mTORC1, which is known to increase the inhibitory effect of the TSC1/TSC2 heterodimer on mTORC1 activity [48,49]. As a result, the combination of metformin and DCA markedly increased REDD1 expression. This result is in line with previous studies reporting that metformin inhibits mTORC1 by AMPK-independent mechanisms [50]. Lastly, we suggest that oxidative stress induced by metformin, DCA, or both played an important role in the potent anticancer effect of metformin and DCA in liver cancer cells. 

There is a difference from previous studies [43]. Previous studies mainly focused on effective suppression of mTORC1 signaling by a combination treatment of conventional chemotherapy or radiotherapy (DNA/RNA damage), and tyrosine kinase inhibitors (EGFR inhibition) with metformin, thereby increasing anticancer effects. In this study, two drugs of DCA and metformin were focused on inhibition of main energy production pathways such as glycolysis and mitochondrial respiration in liver cancer cells. Metformin increased glycolysis to compensate energy depletion after inhibition of mitochondrial respiration in this study. Increased glycolysis can play a role to activate signaling related to epithelial-mesenchymal transition or chemo-resistance in previous studies [51]. Thus, it is important to control increasing glycolysis rates by DCA after metformin treatment in liver cancer cells. In a combination treatment of metformin and DCA, ATP production in liver cancer cells was effectively suppressed and at the same time, lactate production was also controlled.

This study has clinical implications by suggesting new agents for treating liver cancer. Previous studies demonstrated that activating PI3K/Akt/mTORC1 signaling is associated with acquired resistance to sorafenib in patients with HCC [52]. Furthermore, activation of the PI3K/Akt/mTORC1 signaling pathway may promote hepatocarcinogenesis and the epithelial-mesenchymal transition [52,53,54]. One study reported that the epithelial-mesenchymal transition is associated with drug resistance, as well as invasive and metastatic properties of cancer cells [55]. In addition, previous studies have determined that depleting intracellular ATP and increasing ROS production attenuate the epithelial-mesenchymal transition and drug resistance [56,57]. Considering the low tumor response rates of currently used molecular targeted therapy (<15%) in liver cancer patients [16], and the importance of PI3K/Akt/mTORC1 signaling, ATP depletion, and oxidative stress during the epithelial-mesenchymal transition in cancer cells, co-treatment of metformin and DCA targeting mTORC1 might be an effective alternative for liver cancer patients who have failed treatment of tyrosine kinase inhibitors or who developed intra/extra-metastasis. 

In conclusion, liver cancer cells became more glycolytic in response to the metformin treatment. Co-treatment with metformin and DCA can suppress the growth of liver cancer by blocking energy, inhibiting mTORC1 expression, and increasing oxidative stress.. This study suggests a new therapy for liver cancer in the future. Further studies are required to determine the effectiveness of co-treatment with metformin and DCA in other animal models, particularly with an efficient drug delivery system bearing the two agents.

## 4. Materials and Methods

### 4.1. Cell Lines and Chemicals

The HepG2 and PLC/PRF5 human liver cancer cell lines for in vitro studies and the MH-134 murine hepatoma cell line for in vivo studies were purchased from the Korean Cell Line Bank. Both cell lines were maintained in Dulbecco’s modified Eagle’s medium with 10% heat-inactivated fetal bovine serum, 100 U/mL penicillin G, and 100 µg/mL streptomycin at 37 °C in a 5% CO_2_ incubator. Human primary hepatocytes were purchased from Nexel (Hepatosight^®^-S H-002, Seoul, Korea), and plated on collagen 1 (Sigma Aldrich)-coated 12-well plates and maintained in hepatocyte culture medium provided by the manufacturer (Hepatosight^®^-S H-002, Seoul, Korea). Metformin and DCA were prepared freshly and added to the cell cultures at the indicated concentrations for up to 24 h. The AMPK inhibitor, compound C (CC) was purchased from Sigma-Aldrich (St. Louis, MO, USA, Cat. No. 171260), and was dissolved in DMSO and used at the 20 µM concentration for each specific study. When treatment with metformin in the HepG2 and PLC/PRF5 cells, CC was pretreated for 1 h with 20 µM CC followed by treatment with 10 mM metformin

### 4.2. Cell Viability Assay

Cell viability was measured using the MTT reagent (Sigma-Aldrich, St. Louis, MO, USA) dissolved in PBS (0.5 mg/mL). On the day of measurement, the medium was replaced with MTT medium and incubated for 4 h at 37 °C in the dark. After removing the incubation medium, the formazan crystals were dissolved in DMSO for 1 h. Reduced MTT was quantified by measuring the light absorbance at 540 nm using the Spectra MAX 190 microplate reader (Molecular Devices, Sunnyvale, CA, USA). Untreated cells were considered the control (100% cell survival).

### 4.3. Western Blot Analysis

Cells were lysed using protein extraction buffer (iNtRON Biotechnology, Seongnam-Si, Gyeonggi-do, Korea) and the protein concentrations of the cell lysates were assessed with the BCA protein Assay (Thermo Scientific, Pittsburgh, PA, USA). A 20–30 µg aliquot of protein from the whole cell lysates was separated by 8–12% sodium dodecyl sulfate-polyacrylamide gel electrophoresis and transferred to a PVDF membrane. After being blocked with 5% skim milk for 1 h, the membranes were probed with primary antibodies (1:1000 dilution) against poly-ADP-ribose polymerase (PARP), cleaved PARP, β-actin, pyruvate dehydrogenase (PDH)-E1α, p-PDH-E1α, p-phosphatidylinositol-3-kinase (PI3K), anti-PI3K, serine/threonine kinase (Akt), p-Akt, mammalian target of rapamycin complex 1 (mTORC1), p-mTORC1, p-AMPKα (Thr172), AMPKα, regulated in development and DNA damage responses 1 (REDD1), p-70 S6 kinase (S6K1) (Thr389), S6K1, 4E-binding protein 1 (4EBP1), p-4EBP1, acetyl-CoA carboxylase (p-ACC), p-ACC at 4 °C overnight (Cell Signaling Technology, Danvers, MA, USA). This was followed by incubation with a horseradish peroxidase-conjugated secondary antibody (1:10,000 dilution) for 2 h at room temperature. The antibody-reactive band was detected by chemiluminescence (Bio-Rad Laboratories, Hercules, CA, USA).

### 4.4. Flow Cytometry with Annexin V-FITC Staining

Apoptotic analyses were also performed by flowcytometry, using a FACScalibur system (Becton Dickinson Biosciences, San Diego, CA, USA). Cells were co-treated with metformin and DCA for 24 h. After treatment, cells were collected by trypsinization, washed twice with ice-cold PBS, and re-suspended in 0.5 mL of annexin V-FITC solution for 15 min in dark at room temperature. After staining, cells were injected through flow cytometer and analyzed for FITC fluorescent signals. Apoptotic cells were analyzed by quadrant statistics on the propidium iodide-negative and annexin V-positive cells. Means ± SD were calculated for the cell populations from three replicate data.

### 4.5. Extracellular Lactate Assay and Intracellular ATP Assay

Lactate production was determined using the EZ-Lactate Assay Kit (DoGenBio, Seoul, Korea). The cells were treated with metformin and DCA for 24 h. The cell culture medium was collected and lactate levels were assayed by colorimetric detection at 450 nm according to the manufacturer’s instructions. The level of ATP production was determined using the EZ-ATP Assay Kit. After a 24 h treatment, the HepG2 and PLC/PRF5 cells were harvested and assayed via colorimetric detection at 570 nm using an enzyme-linked immunosorbent assay (ELISA) spectrophotometer. Absorbance was normalized to protein concentration.

### 4.6. Assessment of Intracellular and Mitochondrial Reactive Oxygen Species Generation

Dihydroethidium (DHE) is converted by superoxide into red fluorescent ethidium (Sigma-Aldrich, St. Louis, MO, USA). Intracellular ROS levels were detected using 5 µM DHE in a 37 °C incubator for 30 min under a dark condition. Subsequently, the cells were harvested after a 1 h incubation with 0.1 N NaOH. The intensity of DHE cellular fluorescence was determined at 590 nm excitation and 650 nm emission using the Spectra Max M2 ELISA plate reader (Molecular Devices, Sunnyvale, CA, USA). The experiments were performed in triplicate and the final values were normalized to the total number of cells.

MitoSOX-based assays (Invitrogen, Waltham, MA, USA) were also used to detect mitochondrial reactive oxygen species. Flow cytometric protocol involving the use of 5 μM of MitoSOX detected mitochondrial ROS in HepG2 cells with treatment of metformin and/or DCA. The measurement of MitoSOX-derived fluorescence intensity reflected the levels of mitochondrial total ROS. After treatment, cells were stained with dye at 37 °C for 10 min according to the manufacturer’s instruction. Mitochondrial ROS activities were analyzed by flow cytometry. 

### 4.7. In Vivo Subcutaneous Xenograft Model

Briefly, MH-134 cells (5 × 10^7^ cells per mouse) were subcutaneously transplanted into the flanks of C3H mice (*n* = 6 per group). The tumor volume was measured using a Vernier caliper and calculated as [length × (width)^2^]/2. When the tumor volume reached a size of approximately 200 mm^3^, mice were treated orally with saline or metformin (200 mg per kg) or combination of metformin (200 mg per kg) and DCA (100 mg per kg) on a daily basis. The length and width of each nodule were measured every day for 15 days. To confirm tumor apoptosis, terminal deoxynucleotidyl transferase dUTP nick end labeling (TUNEL) staining was performed. Tumor specimens were fixed in 4% formaldehyde and embedded in paraffin. For immunohistochemical (IHC) analyses, specimens were cut into 4 μm sections. IHC staining was performed using the anti-mouse Ki-67 antibody (Abcam, Cambridge, UK) at a 1:3000 dilution rate, anti-cyclin D1 antibody (Cell Signaling Technology, Beverly, MA, USA) at a 1:50 dilution rate, anti-mouse proliferating cell nuclear antigen antibody (Thermo Scientific, Pittsburgh, PA, USA) at a 1:5000 dilution rate, and 4-hydroxynonenal (HNE) antibody (LSBio, Seattle, WA, USA) at a 1:1000 dilution rate. All protocols for the animal experiments were reviewed and approved by the Institutional Animal Care and Use Committee (No. IACUC200170, ethic approval date: 19 November 2020). All animal procedures were in accordance with the “Guide for the Care and Use of Laboratory Animals” issued by the Institute of Laboratory Animal Resources Commission on Life Science, US National Research Council.

### 4.8. Statistical Analysis

All results were reported as the means ± standard deviations (SD). The paired Student’s *t*-test was applied to detect differences between the two groups. All *p*-values <0.05 were considered significant. All statistical analyses were performed using SPSS version 18.0 (IBM, Armonk, NY, USA).

## Data Availability

Data are contained within the article and supplementary material.

## Supplementary Materials

Supplementary materials can be found at https://www.mdpi.com/article/10.3390/ijms221810027/s1.
