# Supplementary material for: Metformin and Dichloroacetate Suppress Proliferation of Liver Cancer Cells by Inhibiting mTOR Complex 1
Source: Int J Mol Sci. 2021 Sep 17;22(18):10027. doi: 10.3390/ijms221810027 (PMC8467948; doi:10.3390/ijms221810027)
Supplement: Supplementary file 1 [file ijms-22-10027-s001.zip › ijms-1331679-supplementary.pdf]

Supplementary Materials

Metformin and Dichloroacetate Suppress Proliferation of Liver Cancer Cells by Inhibiting mTOR Complex 1

Tae Suk Kim [1], Minjong Lee [2,3,*], Minji Park [2,3,4], Sae Yun Kim [5], Min Suk Shim [6], Chea Yeon Lee [1], Dae Hee Choi [1] and Yuri Cho [4,7,*]

1 Department of Internal Medicine, Kangwon National University Hospital, Kangwon National University School of Medicine, Chuncheon 24341, Korea; greatstone@kangwon.ac.kr (T.S.K.); qnwia@naver.com (C.Y.L.); dhchoi-md@kangwon.ac.kr (D.H.C.)
2 Department of Internal Medicine, Ewha Womans University College of Medicine, Seoul 07804, Korea; jewelbox6@hanmail.net
3 Department of Internal Medicine, Ewha Womans University Medical Center, Seoul 07804, Korea
4 Department of Internal Medicine, CHA Gangnam Medical Center, CHA University School of Medicine, Seoul 06135, Korea
5 Department of Pediatrics, College of Medicine, The Catholic University of Korea, Seoul 06591, Korea; sysmile@gmail.com
6 Division of Bioengineering, Incheon National University, Incheon 22012, Korea; msshim@inu.ac.kr
7 Center for Liver and Pancreatobiliary Cancer, Research Institute and Hospital, National Cancer Center, Goyang 10408, Korea
* Correspondence: minjonglee2@naver.com (M.L.); yuricho@ncc.re.kr (Y.C.); Tel.: +82-2-6986-1671 (M.L.); Tel: +82-31-920-1605 (Y.C.)

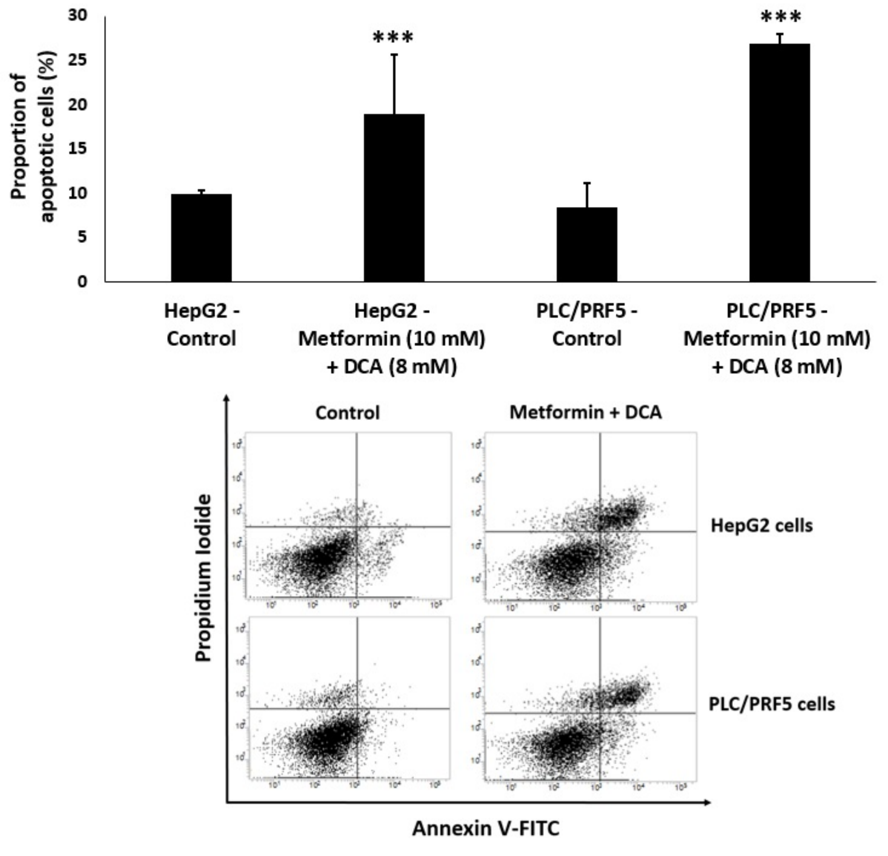

Supplementary Figure S1. Apoptosis rates of HepG2 and PLC/PRF5 cells after a combination treatment of metformin and DCA evaluated by annexin V-FITC staining. The upper panel shows the quantitative result, and the lower panel depicts the proportion of apoptotic cells. Metformin (10 mM) and DCA (8 mM) were treated for 24 hours in each cell. *** $P < 0.001$.

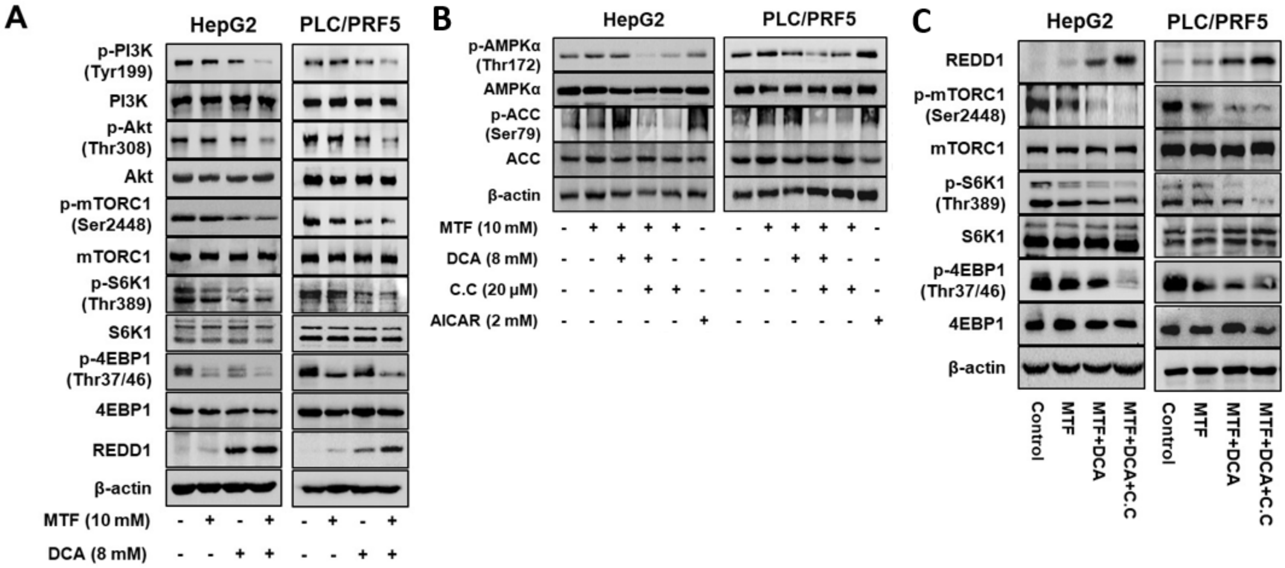

Supplementary Figure S2. Western blotting of PI3K/Akt/mTORC1 signaling when co-treatment with metformin and DCA suppressed mTOC1 activity in HepG2 and PLC/PRF5 cells.

This figure is a supplementary data of western blotting for Figure 4. Each (A), (B), and (C) panel showed change of protein expression in PI3K/Akt/mTORC1 signaling. Cells were exposed to 10 mM metformin, 8 mM DCA, or both for 24 hours. (A) Co-treatment with metformin and DCA significantly suppressed PI3K/Akt/mTORC1 signaling in liver cancer cells compared to the control. (B) Co-treatment with metformin and DCA stimulated p-ACC expression as an AMPK effector in liver cancer cells compared to the control. (C) Metformin and DCA effectively induced REDD1 expression.

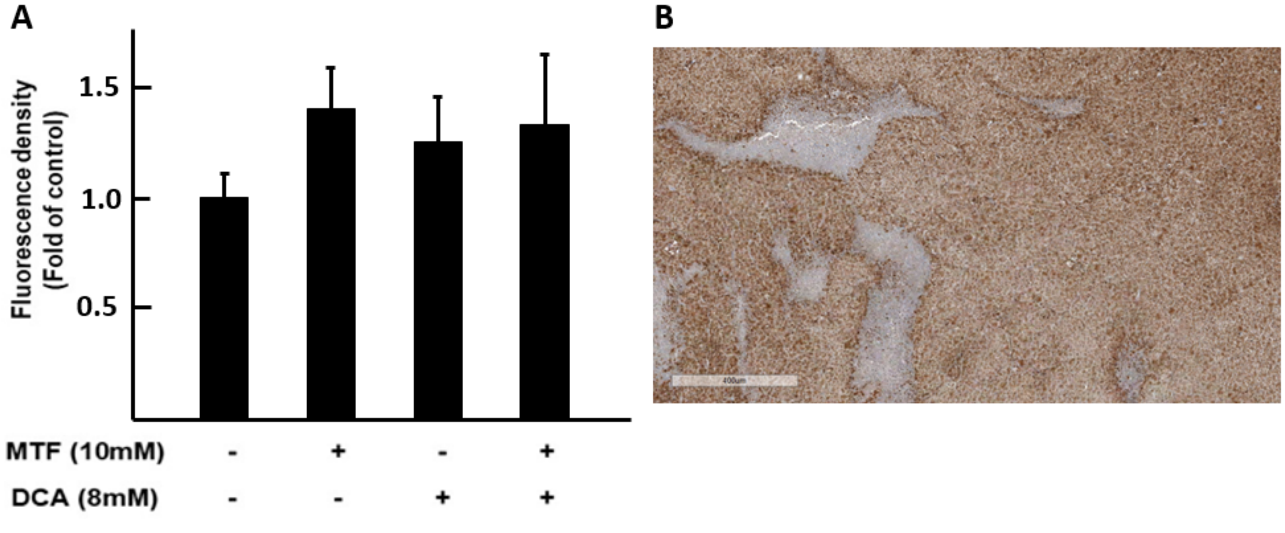

Supplementary Figure S3. Changes of mitochondrial reactive oxygen species in HepG2 cells treated with metformin, DCA, or both for 1 hour. (**A**) Co-treatment with metformin and DCA significantly increased mitochondrial reactive oxygen species (ROS) production in HepG2 cells compared to the control. (**B**) In tumor tissue, 4-hydroxynonenal (4-HNE) in the group treated with metformin and DCA was highly expressed (×100).

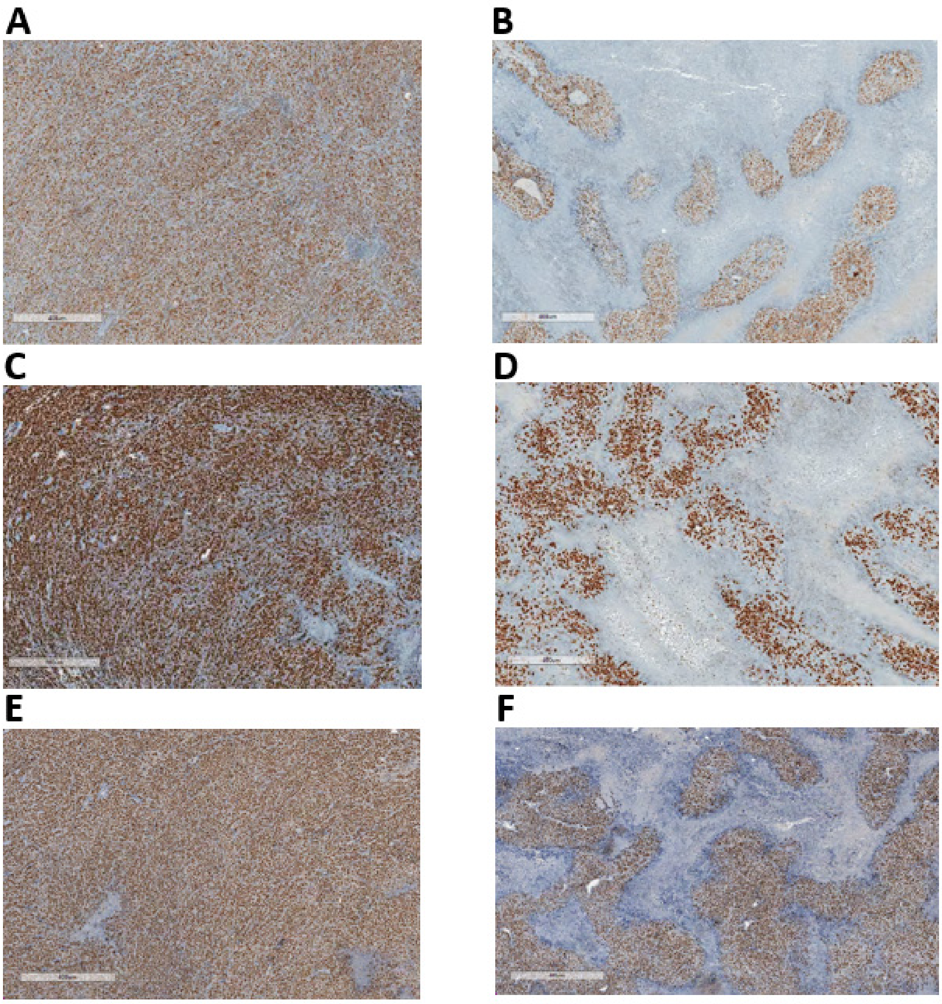

Supplementary Figure S4. Expression of cyclin D1, Ki-67 protein, and proliferating cell nuclear antigen (PCNA) was decreased in tumor tissues in the group treated with metformin and DCA compared to the control group. (**A**) Expression of cyclin D1 protein in the control group. (**B**) Expression of cyclin D1 protein in the group treated with metformin and DCA. (**C**) Expression of Ki-67 protein in the control group. (**D**) Expression of Ki-67 protein in the group treated with metformin and DCA. (**E**) Expression of PCNA in the control group. (**F**) Expression of PCNA in the group treated with metformin and DCA.

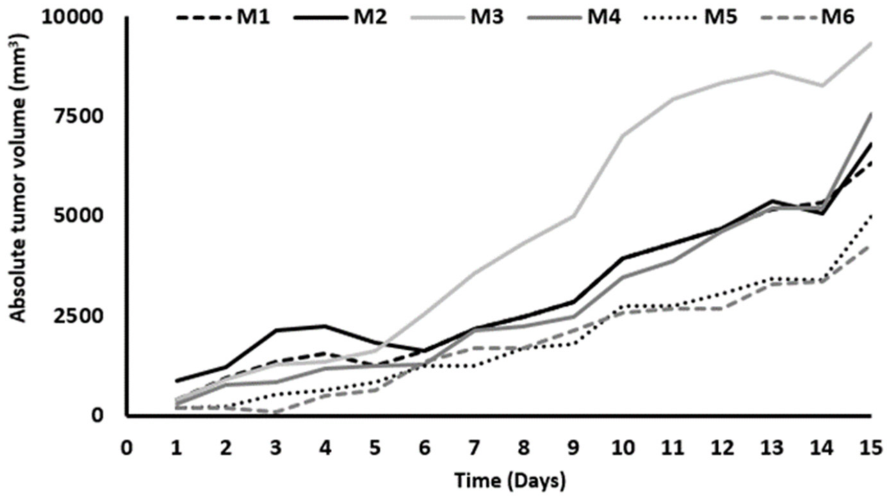

Supplementary Figure S5. The individual changes of tumor volume in the combination treatment group treated with metformin and DCA over time.
